# Author’s Reply to: Comment on “Web-Based Measure of Life Events Using Computerized Life Events and Assessment Record (CLEAR): Preliminary Cross-Sectional Study of Reliability, Validity, and Association With Depression”: Validity and Methodological Issues

**DOI:** 10.2196/15434

**Published:** 2020-05-21

**Authors:** Antonia Bifulco

**Affiliations:** 1 Middlesex University London United Kingdom

**Keywords:** life events, validity, depression, measure

I thank the editors of *JMIR Mental Health* for the opportunity to respond to the Letter to the Editor [1] critiquing the methodology of the Computerised Online Life events Record (CLEAR) [2] published in this journal. While appreciating the critique, it expands on only one of the validity findings, comparing CLEAR with a life event interview and checklist questionnaire. There are significant points not outlined in the letter [1] and these are addressed here. Without questioning the additional statistical analyses provided by the sensitivity and specificity, the conclusion that this is unacceptable for a “diagnostic tool” is refuted. CLEAR is not a clinical diagnostic tool but owing to its high predictive validity with regard to depression and superiority to the checklist life events test (LTE-Q) [3], it retains value as a method for identifying environmental stressors related to clinical models.

The ambition of the CLEAR measure design needs to be understood. It attempts to capture similar data to those established in the intensive, contextualized, face-to-face, semistructured Life Events and Difficulties Interview (LEDS) [4] by using very detailed, interactive, time-based online self-report material. This is both complex and novel. Such conversion of the interview has never previously been undertaken despite various attempts at further structuring the interview for computerization [5]. The value of CLEAR lies in the large amount of event information and context generated, both quantitative and qualitative, and the preprogramming of data to produce both derived risk factors and partly analyzed data that are easily downloadable for analysis. CLEAR also provides a tailored feedback report for respondents. Besides identifying events, it can specify life event characteristics—not only their usual domain categorizations, but also their severity of threat/unpleasantness; focus of event; and characteristics such as loss, danger, and humiliation. It also identifies positivity of events and related characteristics (eg, relief, goal achievement). This is in addition to the timing of events and those stressors constituting longer-term problems. No checklist can provide such detailed data. Although interviews can provide such data, CLEAR provides very large savings in time and cost, expertise of investigators, and respondent convenience in large-scale data collection. Therefore, the balance of the reported lower sensitivity of the tool to interviews (ie, in missing some life events) needs to be considered in relation to the higher specificity with associated richness of event characteristics and the associated increased prediction of depression [2,4].

Further points in response to this paper are given below:

- The authors of the letter point out that the sensitivity of CLEAR is weaker than its specificity. This is due, in large part, to the different modes of administering the tools. The results show that an interview (with the investigator asking probing questions) elicits more life events than individuals identify online without such personalized probes, thus yielding lower sensitivity. Further qualitative investigation in the study showed that this was largely because a trained investigator identified more stages in a developing event crisis in the same category—the CLEAR tool tended to combine these into single events. However, all key crisis events were reflected in CLEAR when compared qualitatively with the interview. Therefore, high predictive validity for depression was retained. This limitation is more pronounced in a checklist approach where only one event per category applies, with no differentiation of phases in a developing crisis possible. The online CLEAR is superior to the checklist with better predictive validity.
- There was higher validity found in the group studied, which included midlife clinical and case control individuals and young adult students. Midlife individuals responded more accurately than the students in interview accounts. Because of the restricted numbers in the validation exercise, due to interview time taken, both age groups were combined. We believe young people rushed through the exercise more, and even when interviewed, had a more confused reporting style. We are examining ways of further structuring the measure to avoid this in younger age groups.
- CLEAR severe events significantly differentiate the matched depression and control groups as hypothesized [2].
- The authors do not address predictive validity in their paper. CLEAR severe life events are significantly related to depression, indicating high predictive validity [6]. Further investigation showed that this increased from any severe life events (odds ratio [OR]=3.59) to those involving humiliation (OR=6.38) or trauma (OR=5.83). In comparison, the LTE-Q checklist events in this study do not predict depression [2].

However, we do take messages from the letter by Rahmani and colleagues [1] and are seeking to increase the validity of CLEAR as compared with the LEDS interview from the observations and qualitative aspects taken from the analyses. This involves providing more structured options on the menu of life events checked. We believe having an online tool such as CLEAR will enable wider investigation of life events and their role in depression for both research and clinical practice.

## Abbreviations

CLEAR: Computerised Online Life events Record
LEDS: Life Events and Difficulties Interview
LTE-Q: checklist life events test
